# Analysis of factors influencing bronchiectasis patients with active pulmonary tuberculosis and development of a nomogram prediction model

**DOI:** 10.3389/fmed.2024.1457048

**Published:** 2024-11-08

**Authors:** Yitian Yang, Lianfang Du, Weilong Ye, Weifeng Liao, Zhenzhen Zheng, Xiaoxi Lin, Feiju Chen, Jingjing Pan, Bainian Chen, Riken Chen, Weimin Yao

**Affiliations:** The Second Affiliated Hospital of Guangdong Medical University, Zhanjiang, Guangdong, China

**Keywords:** active pulmonary tuberculosis, bronchiectasis, nomogram, prediction model, infection

## Abstract

**Background:**

To identify the risk factors for bronchiectasis patients with active pulmonary tuberculosis (APTB) and to develop a predictive nomogram model for estimating the risk of APTB in bronchiectasis patients.

**Methods:**

A retrospective cohort study was conducted on 16,750 bronchiectasis patients hospitalized at the Affiliated Hospital of Guangdong Medical University and the Second Affiliated Hospital of Guangdong Medical University between January 2019 and December 2023. The 390 patients with APTB were classified as the case group, while 818 patients were randomly sampled by computer at a 1:20 ratio from the 16,360 patients with other infections to serve as the control group. Relevant indicators potentially leading to APTB in bronchiectasis patients were collected. Patients were categorized into APTB and inactive pulmonary tuberculosis (IPTB) groups based on the presence of tuberculosis. The general characteristics of both groups were compared. Variables were screened using the least absolute shrinkage and selection operator (LASSO) analysis, followed by multivariate logistic regression analysis. A nomogram model was established based on the analysis results. The model’s predictive performance was evaluated using calibration curves, C-index, and ROC curves, and internal validation was performed using the bootstrap method.

**Results:**

LASSO analysis identified 28 potential risk factors. Multivariate analysis showed that age, gender, TC, ALB, MCV, FIB, PDW, LYM, hemoptysis, and hypertension are independent risk factors for bronchiectasis patients with APTB (*p* < 0.05). The nomogram demonstrated strong calibration and discrimination, with a C-index of 0.745 (95% CI: 0.715–0.775) and an AUC of 0.744 for the ROC curve. Internal validation using the bootstrap method produced a C-index of 0.738, further confirming the model’s robustness.

**Conclusion:**

The nomogram model, developed using common clinical serological characteristics, holds significant clinical value for assessing the risk of APTB in bronchiectasis patients.

## 1 Introduction

Bronchiectasis (BE) is a chronic respiratory disease characterized by abnormal and irreversible dilation and distortion of the small airways, which leads to bacterial colonization in the damaged airways and results in chronic cough and sputum production (1). The development of bronchiectasis invariably involves inflammation in the bronchial walls, with most patients exhibiting mixed inflammation predominantly involving neutrophils, though some patients also show increased numbers of eosinophils and monocytes (2, 3). Inflammation has multiple sources, in most cases, it is caused by pathogenic microorganisms (usually bacteria and mycobacteria) (4, 5). Neutrophils play a critical role in the pathogenesis of bronchiectasis by contributing to structural alterations in the bronchial wall, causing damage that leads to bronchial dilation (6).

In Asia, the prevalence of bronchiectasis is significantly higher than in Western countries (7) and APTB is one of the main causes of bronchiectasis (8, 9). According to an analysis of data from the Korean Bronchiectasis Audit and Research Collaboration cohort, APTB is the most common non-idiopathic cause of bronchiectasis, followed by pulmonary infections, asthma, non-tuberculous mycobacterial (NTM) infections, and COPD (10). Post-tuberculosis bronchiectasis prevalence ranges from 35 to 86% based on radiological findings (11). APTB and bronchiectasis are interrelated, and bronchiectasis may occur during tuberculosis or as a sequela of the disease (12). A study has shown that differences in severity and mortality between bronchiectasis patients with APTB and those with bronchiectasis due to other causes (13). Currently, literature reports that there are significant differences (*p* < 0.05) in serological indicators such as adenosine deaminase, lactate dehydrogenase (14), platelet count, thrombin time, D-dimer (15), and fibrinogen (16) between patients with APTB and healthy individuals. Compared to patients without APTB, patients with APTB show significant differences (*p* < 0.05) in mean corpuscular volume, erythrocyte sedimentation rate, albumin levels, adenosine deaminase levels, monocyte count to high-density lipoprotein ratio, and high-sensitivity C-reactive protein to lymphocyte count ratio (17). Research has demonstrated that coagulation indicators change following antituberculosis treatment in TB patients, with APTT and Fbg-C levels significantly higher in TB patients compared to healthy individuals (18). This suggests a correlation between coagulation indicators and the APTB.

Currently, there are no reports analyzing the hematological parameters influencing the co-occurrence of bronchiectasis with active pulmonary tuberculosis. This study analyzes the factors affecting patients with bronchiectasis combined with active pulmonary tuberculosis and establishes a predictive model using a nomogram. The nomogram prediction model is simple and practical, allowing for the quantification of the incidence and recurrence of clinical events (19). The aforementioned inflammation and coagulation markers may aid in distinguishing PTB activity. Moreover, these biomarkers are easily accessible, making a scoring system based on them highly applicable in clinical practice, particularly in resource-limited areas. It holds great significance for disease screening.

## 2 Materials and methods

### 2.1 Patients and study design

A retrospective cohort study was conducted on 16,750 bronchiectasis patients hospitalized at the Affiliated Hospital and the Second Affiliated Hospital of Guangdong Medical University from January 2019 to December 2023. Of these, 390 patients were diagnosed with APTB, while 16,360 patients suffered from other bacterial infections (other bacterial infections in the lungs are caused by Gram-negative or Gram-positive bacteria, as confirmed by sputum culture results. These infections are often associated with elevated inflammatory markers such as CRP and procalcitonin, increased neutrophil counts, or symptom improvement following empirical antibiotic treatment, even in the absence of definitive pathogen identification). The 390 patients with APTB were classified as the case group, while 818 patients were randomly sampled by computer at a 1/20 ratio from the 16,360 patients with other infections to serve as the control group. Clinical data were collected for these 1,208 patients, including general information (such as age and gender), and serological characteristics [including: lactate dehydrogenase (LDH), triglycerides (TG), high-density lipoprotein (HDL), low-density lipoprotein (LDL), total cholesterol (TC), albumin (ALB), prothrombin time (PT), activated partial thromboplastin time (APTT), fibrinogen (FIB), thrombin time (TT), monocytes (MON), white blood cells (WBC), lymphocytes (LYM), neutrophils (NEU), red blood cells (RBC), hemoglobin (HGB), red cell distribution width (RDW), mean corpuscular volume (MCV), hematocrit (HCT), platelets (PLT), platelet distribution width (PDW), mean platelet volume (MPV), and plateletcrit (PCT), totaling 36 potential risk factors].

This study was approved by the ethics committees of the Affiliated Hospital of Guangdong Medical University (No: PJKT2024-053) and the Second Affiliated Hospital of Guangdong Medical University (No: PJKT2024-022).

### 2.2 Diagnostic criteria for APTB and BE

Bronchiectasis Diagnosis Criteria: Patients must meet the criteria outlined in the “European Respiratory Society guidelines for the management of adult bronchiectasis” (20), specifically abnormal radiographic changes indicative of bronchiectasis on high-resolution computed tomography (HRCT). APTB Diagnosis Criteria: Patients must meet the criteria specified in the “WHO consolidated guidelines on tuberculosis: Module 3: Diagnosis – Tests for tuberculosis infection” (21), which include a positive sputum acid-fast bacilli smear and effective anti-tuberculosis treatment, or a positive nucleic acid detection for *Mycobacterium tuberculosis* in bronchial alveolar lavage fluid using next-generation sequencing (NGS). Patients with bronchiectasis who develop APTB are defined as those in whom bronchiectasis precedes the onset of pulmonary tuberculosis, meaning that bronchiectasis is not caused by tuberculosis.

Exclusion Criteria: (1) Patients with diseases of vital organs such as the liver or kidneys. (2) Patients with concomitant human immunodeficiency virus (HIV) infection and cellular immunodeficiency. (3) Patients with concomitant malignant tumors, autoimmune diseases, or chronic viral infections (such as hepatitis B or hepatitis C). (4) Patients with severe circulatory dysfunction. (5) Patients who have recently received immunostimulant or immunosuppressant therapy. (6) Extrapulmonary infections.

### 2.3 Statistical analysis

Statistical analyses were performed using SPSS 20.0 software. Count data were expressed as *n* (%), and comparisons between groups were made using the *χ^2^* test. Normally distributed continuous data were expressed as mean ± standard deviation ( $\bar{x}$ *±s*), and comparisons between two groups were made using the independent sample *t*-test. Non-normally distributed continuous data were expressed as median [*M (Q1, Q3)*], and comparisons between groups were made using the rank-sum test.

The Least Absolute Shrinkage and Selection Operator (LASSO) regression analysis was employed to identify potential risk factors. Multivariate logistic regression analysis was then conducted on the identified variables to further screen for independent risk factors for bronchiectasis with APTB. The nomogram model was constructed using the “rms” package in R software. The receiver operating characteristic (ROC) curve was plotted to evaluate the model’s discrimination ability, and a calibration curve was drawn to assess and refine the model. The Bootstrap method was used for internal validation. Finally, decision curve analysis (DCA) was performed to evaluate the clinical utility of the model. All analyses were performed using R software (version 4.3.3). *p* < 0.05 was considered statistically significant.

## 3 Results

### 3.1 Study populations and univariate analysis results

The case group consisted of 390 patients with APTB, while the control group included 818 patients with other infections. Compared to the control group, the case group showed significantly higher values of LDH, TG, PT, APTT, FIB, TT, MON, WBC, NEU, RDW, PLT and PDW, whereas HDL, LDL, TC, ALB, LYM, HGB, MCV and MPV were significantly lower (*p* < 0.05) (Table 1).

**Table 1:** Baseline characteristics and univariate analysis results.

Variables	APTB (*n* = 390)	Other infections (*n* = 818)	*X*^2^/*Z*	*P*
Age				
> = 65	220 (56.4)	448 (54.8)	0.288	0.591
<65	170 (43.6)	370 (45.2)		
Gender				
Man	254 (65.1)	352 (43)	51.577	<0.001
Woman	136 (34.9)	466 (57)		
Smoking				
Yes	64 (16.4)	85 (10.4)	8.848	<0.001
No	326 (83.6)	733 (89.6)		
Drinking				
Yes	30 (7.7)	29 (3.5)	9.777	0.002
No	360 (92.3)	789 (96.5)		
Low fever				
Yes	134 (34.4)	169 (20.7)	26.373	<0.001
No	256 (65.6)	649 (79.3)		
Night sweat				
Yes	7 (1.8)	5 (0.6)	2.655	0.103
No	383 (98.2)	813 (99.4)		
Cough				
Yes	334 (85.6)	765 (93.5)	19.975	<0.001
No	56 (14.4)	53 (6.5)		
Sputum				
Yes	314 (80.5)	729 (89.1)	16.589	<0.001
No	76 (19.5)	89 (10.9)		
Haemoptysis				
Yes	72 (18.5)	312 (38.1)	47.172	<0.001
No	318 (81.5)	506 (61.9)		
Tachypnoea				
Yes	179 (45.9)	399 (48.8)	0.878	0.349
No	211 (54.1)	419 (51.2)		
Hypertension				
Yes	53 (13.6)	155 (18.9)	5.321	0.021
No	337 (86.4)	663 (81.1)		
Diabetes				
Yes	48 (12.3)	46 (5.6)	16.443	<0.001
No	342 (87.7)	772 (94.4)		
CHD				
Yes	21 (5.4)	56 (6.8)	0.945	0.331
No	369 (94.6)	762 (93.2)		
LDH	190.00 (163.00, 224.80)	174.90 (153.00, 204.00)	−5.189	<0.001
TG	0.95 (0.76, 1.25)	0.89 (0.68, 1.21)	−2.383	0.017
HDL	1.10 (0.86, 1.35)	1.32 (1.07, 1.60)	−9.272	<0.001
LDL	2.42 (1.91, 2.96)	2.63 (2.03, 3.19)	−3.662	<0.001
TC	4.05 (3.33, 4.69)	4.46 (3.71, 5.18)	−6.516	<0.001
ALB	34.90 (30.65, 39.20)	39.50 (36.08, 42.40)	−11.315	<0.001
PT	13.20 (12.15, 14.20)	12.70 (11.80, 13.50)	−5.274	<0.001
APTT	34.70 (28.35, 39.95)	31.10 (26.50, 36.50)	−6.242	<0.001
FIB	4.42 (3.77, 5.43)	3.73 (3.06, 4.58)	−9.149	<0.001
TT	16.40 (13.95, 17.75)	15.80 (13.60, 17.30)	−2.742	<0.001
MON	0.64 (0.47, 0.88)	0.56 (0.42, 0.73)	−4.52	<0.001
WBC	7.95 (6.15, 10.36)	7.24 (5.85, 9.28)	−3.629	<0.001
LYM	1.14 (0.72, 1.59)	1.53 (1.08, 2.06)	−9.198	<0.001
NEU	5.77 (4.15, 8.17)	4.74 (3.46, 6.84)	−6.101	<0.001
RBC	4.22 (3.79, 4.63)	4.23 (3.91, 4.67)	−1.195	0.232
HGB	120.00 (106.00, 134.00)	125.00 (114.00, 136.00)	−4.568	<0.001
HCT	31.70 (0.40, 37.85)	33.90 (0.40, 39.00)	−1.718	0.086
MCV	87.60 (82.40, 91.70)	89.80 (86.17, 93.00)	−5.236	<0.001
RDW	13.60 (12.80, 14.95)	13.00 (12.40, 13.90)	−7.419	<0.001
PLT	264.00 (208.50, 325.50)	241.00 (198.15, 295.00)	−4.018	<0.001
MPV	9.10 (8.30, 9.80)	9.50 (8.90, 10.10)	−5.529	<0.001
PCT	0.23 (0.19, 0.29)	0.23 (0.19, 0.27)	−1.803	0.071
PDW	12.40 (9.50, 15.80)	10.95 (9.60, 15.30)	−2.373	0.018

CHD, coronary heart disease; LDH, lactate dehydrogenase; TG, triglycerides; HDL, high-density lipoprotein; LDL, low-density lipoprotein; TC, total cholesterol; ALB, albumin; PT, prothrombin time; APTT, activated partial thromboplastin time; FIB, fibrinogen; TT, thrombin time; MON, monocytes; WBC, white blood cells; LYM, lymphocytes; NEU, neutrophils; RBC, red blood cells; HGB, hemoglobin; HCT, hematocrit; MCV, mean corpuscular volume; RDW, red cell distribution width; PLT, platelets; MPV, mean platelet volume; PCT, plateletcrit; PDW, platelet distribution width.

### 3.2 Screening potential risk factors

**Figure 1:**
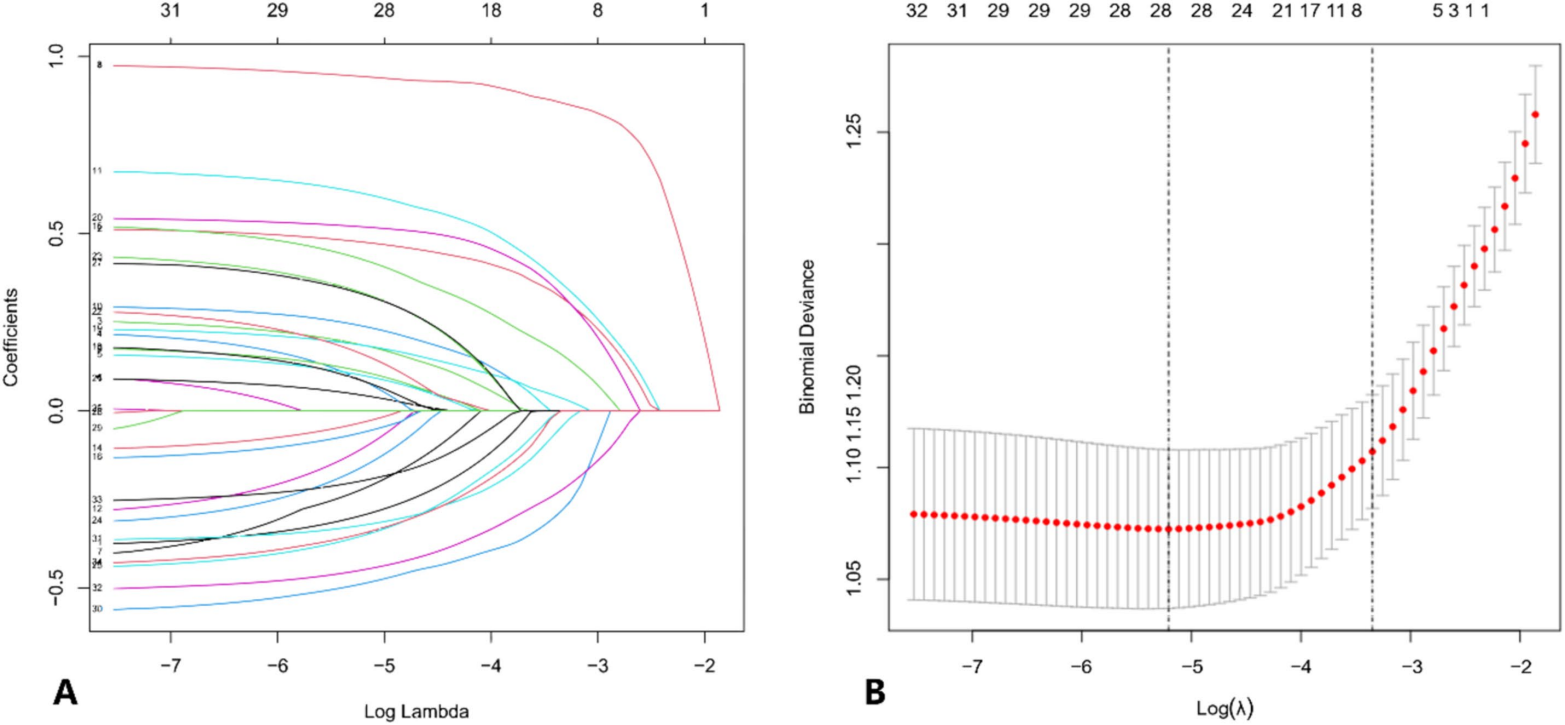
**(A)** Distribution of LASSO coefficients for the risk characteristics. The coefficient profile is plotted against the log (*λ*) sequence. The optimal lambda results in 28 features with non-zero coefficients. **(B)** Selection of the optimal parameters (lambda, *λ*) for the LASSO model using five-fold cross-validation. The binomial deviance curve is plotted against log (lambda). Dotted vertical lines are drawn at the optimal values using the minimum criterion and the 1-standard error (1-SE) criterion.

Patients with concomitant APTB were taken as the dependent variable, while serological and other characteristics were considered as independent variables. To account for multicollinearity among the variables, the optimal penalty coefficient *λ* was determined through five-fold cross-validation in the LASSO regression model. This process ultimately identified 28 potential risk factors: age, gender, LDH, TG, HDL, TC, ALB, PT, APTT, FIB, TT, MON, WBC, LYM, NEU, HCT, MCV, RDW, PLT, MPV, PCT, PDW, drinking, cough, vomica, hemoptysis, polypnea, and hypertension (*p* < 0.05) (Figure 1).

### 3.3 Multivariate logistic regression analysis to identify independent risk factors

The 28 potential risk factors were included in the multivariate logistic regression model, with independent variables transformed into binary variables. The results showed that age, gender, TC, ALB, MCV, FIB, PDW, LYM, hemoptysis, and hypertension are independent risk factors for the occurrence of APTB in bronchiectasis patients (*p* < 0.05) (Table 2).

**Table 2:** Predictive factors for bronchiectasis with active pulmonary tuberculosis identified by multivariate logistic regression analysis.

Intercept and variable	Prediction model		
	β	Odds ratio (95% CI)	*p*-value
Intercept	−0.60	0.54 (0.31–0.95)	0.033
Age	−0.38	0.68 (0.49–0.93)	0.017
Gender	0.52	1.67 (1.24–2.26)	0.0007
TC	0.24	0.70 (0.49–0.98)	0.041
ALB	0.97	2.65 (1.87–3.75)	4.08e-08
FIB	0.68	1.98 (1.46–2.69)	1.12e-05
LYM	0.53	1.69 (1.16–2.47)	0.006
MCV	0.54	1.72 (1.19–2.48)	0.0036
PDW	−0.45	0.64 (0.47–0.86)	0.0032
hemoptysis	−0.51	0.60 (0.42–0.84)	0.0031
hypertension	−0.43	0.65 (0.43–0.96)	0.033

β is the regression coefficient. TC, total cholesterol; ALB, albumin; FIB, fibrinogen; LYM, lymphocytes; MCV, mean corpuscular volume; PDW, platelet distribution width.

### 3.4 Construction of a nomogram model

**Figure 2:**
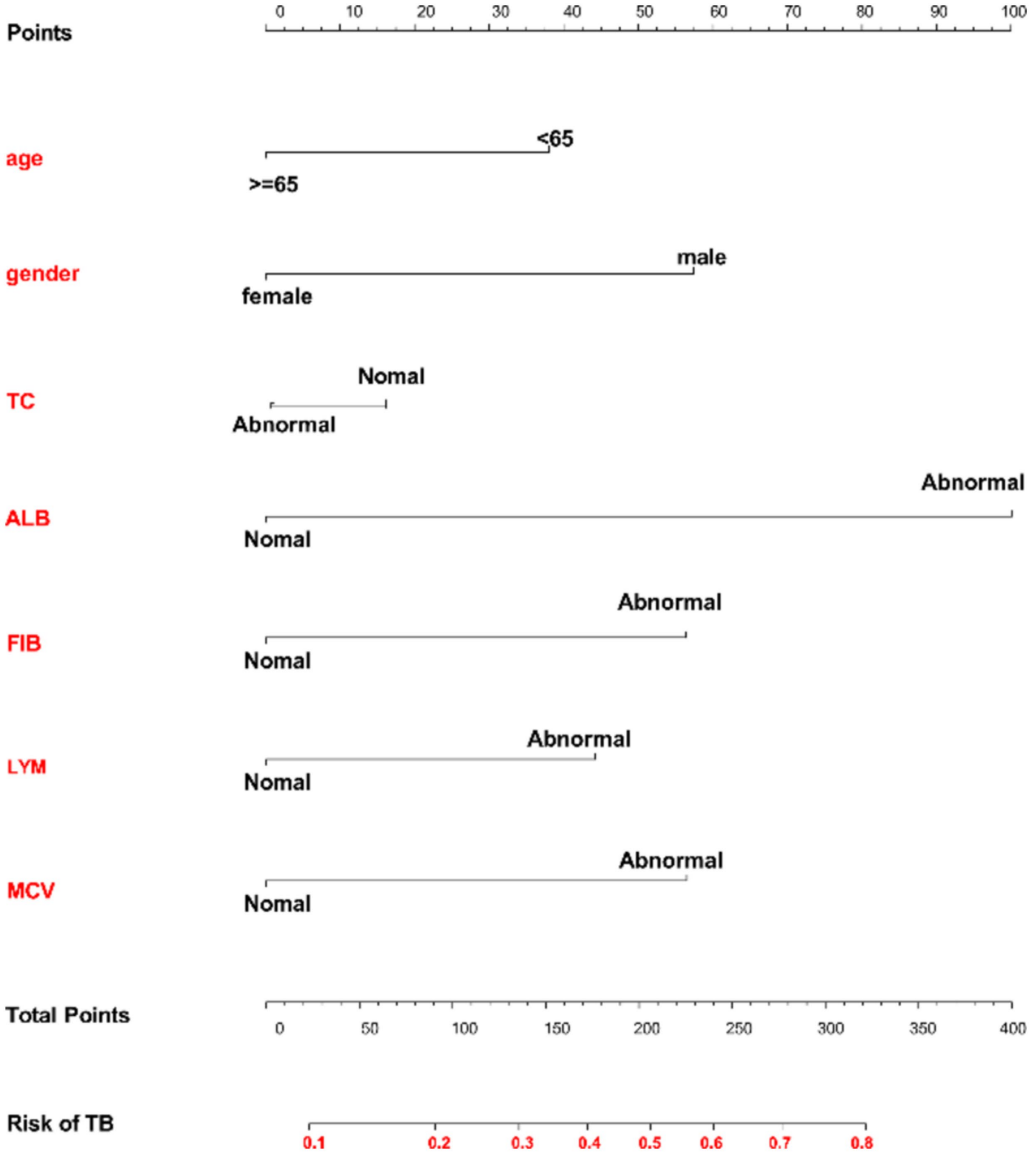
Nomogram model for predicting the risk of concomitant active pulmonary tuberculosis in bronchiectasis patients. Each variable is listed on the left side of the image. Each category for the variables is associated with a certain number of points. These points are found by drawing a vertical line up to the “Points” scale at the top of the nomogram. After calculating the points for each variable based on the patient’s characteristics, you sum up the total points. The total points can be located along the “Total Points” scale at the bottom of the chart. After identifying the total points, you draw a vertical line from the “Total Points” row down to the “Risk of TB” scale. This will give you the predicted probability of developing TB based on the given characteristics.

Based on the results of the multivariate logistic regression analysis and considering clinical practicality (22), we eventually selected seven independent risk factors for the nomogram analysis: age, gender, MCV, TC, ALB, FIB, and LYM. The nomogram evaluation model was constructed using the “rms” package in R software. Each factor was assigned a corresponding score, and the total score was the sum of the scores for each factor. The higher the total score, the higher the probability of bronchiectasis patients developing APTB (Figure 2).

### 3.5 Performance of scoring systems in diagnosing

**Figure 3:**
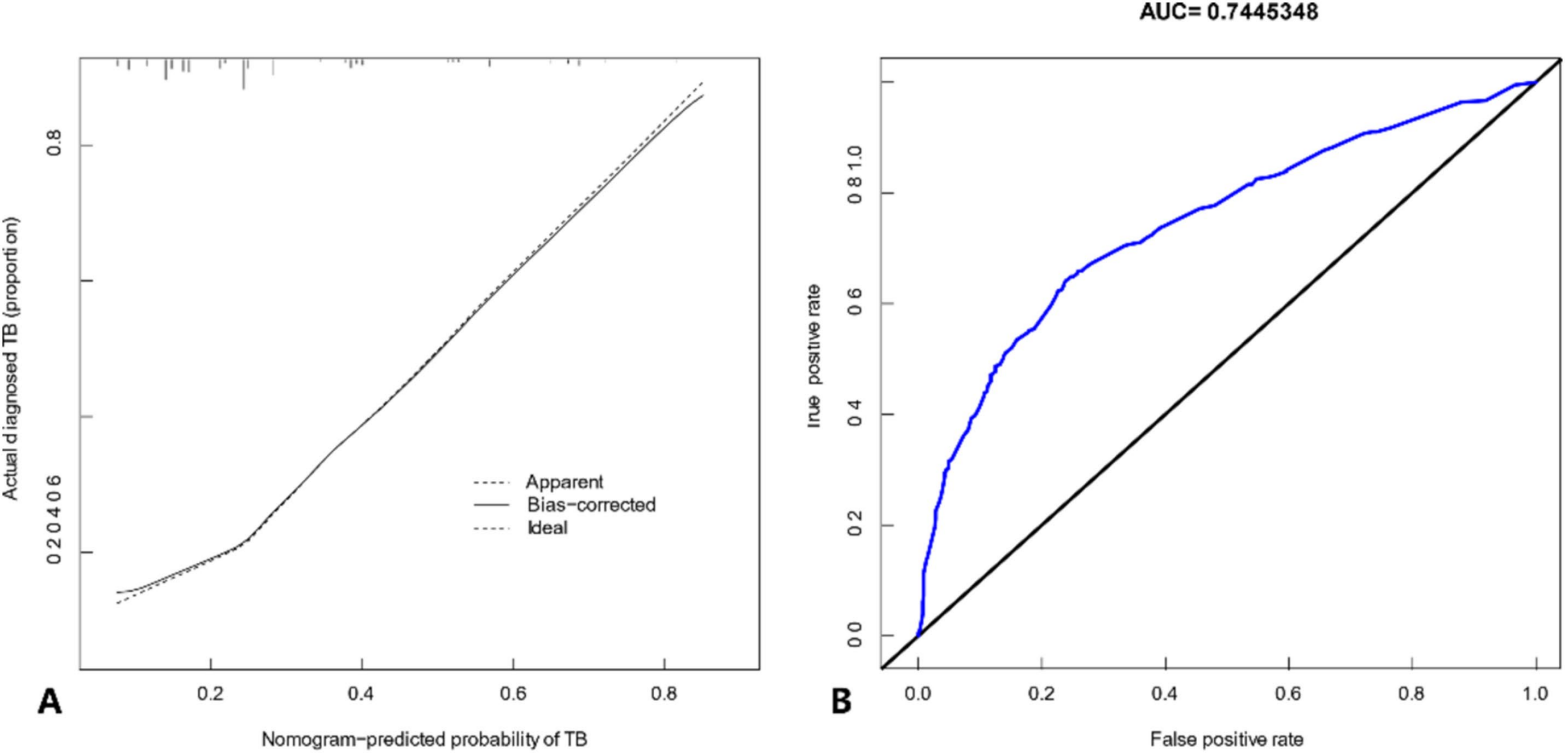
**(A)** Calibration curve. The solid line represents the performance of the nomogram, with closer proximity to the diagonal dashed line indicating better predictive accuracy. **(B)** ROC curve representing the discriminatory power of the model. The dashed line indicates the 95% confidence interval.

The calibration curve indicated strong consistency within this cohort (Figure 3A). The C-index of the nomogram prediction model was 0.745 (95% CI: 0.715–0.775), and internal validation using the Bootstrap method yielded a C-index of 0.738, indicating good discriminative ability. Additionally, the ROC curve generated from the multi-center cohort demonstrated robust predictive value, with an AUC of 0.744 (Figure 3B).

### 3.6 Clinical application value

**Figure 4:**
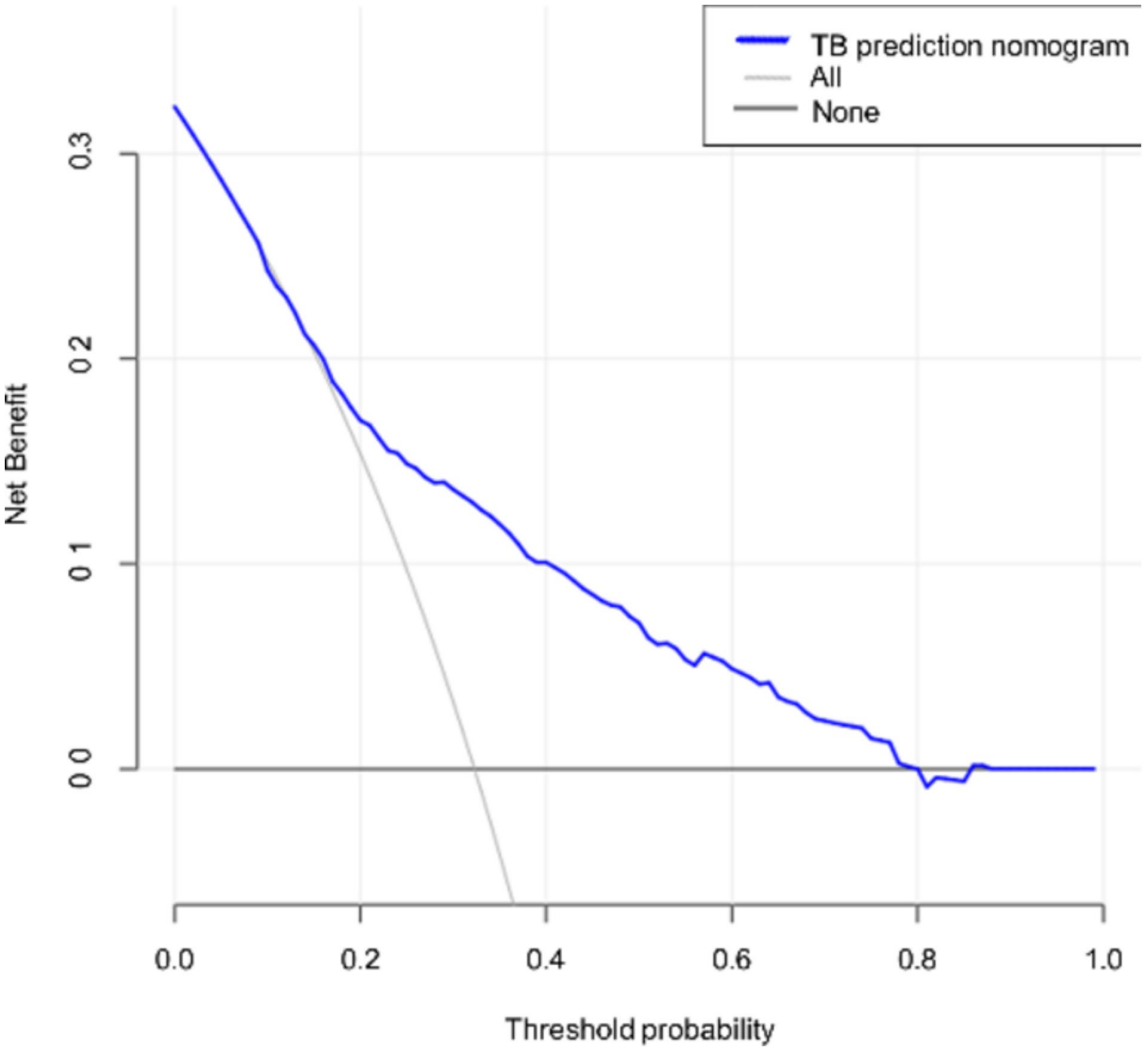
Decision curve for the nomogram. The thin solid line represents the assumption that all patients are in the active tuberculosis phase, while the thick solid line represents the assumption that bronchiectasis patients have other concurrent infections. The decision curve indicates that if the threshold probabilities for patients and doctors are 8 and 79%, respectively, using this nomogram prediction model to assess the risk of concomitant active pulmonary tuberculosis provides greater benefit compared to conventional prediction methods in the current study.

Clinical decision analysis is presented in Figure 4. When the threshold probabilities for patients and doctors are 8 and 79%, respectively, the nomogram model’s prediction of APTB infection risk provides greater benefits.

## 4 Discussion

The APTB is a significant public health issue worldwide, making the timely and accurate identification of suspected cases crucial. APTB and bronchiectasis are interrelated. Our study highlights the risk of APTB in bronchiectasis patients and identifies a total of 28 risk factors through LASSO regression. Further multivariate logistic regression analysis identified 10 independent risk factors: age, gender, TC, ALB, MCV, FIB, PDW, LYM, hemoptysis, and hypertension. Considering clinical practicality, we selected 7 independent risk factors for inclusion in the nomogram analysis: age, gender, MCV, TC, ALB, FIB, and LYM.

Studies have shown that elderly individuals are more susceptible to infectious diseases, particularly respiratory infections (23). The elderly is considered a major reservoir for *Mycobacterium tuberculosis* infection due to their increased susceptibility to new infections and the reactivation of latent *Mycobacterium tuberculosis* infection (24). However, a retrospective cohort study by Abbara et al. (25) indicated that patients aged 65 and above are less likely to exhibit the “classic” clinical or radiological features of APTB, which may lead to delays between symptom onset and diagnosis. Our nomogram prediction model suggests a lower prevalence of APTB in elderly individuals, which may be attributed to decreased immune sensitivity in this population. Van Zyl Smit et al. (26) provided evidence that more men than women are infected with APTB (26). However, some scientists believe that the incidence of APTB infection in women may be underestimated in developing regions (27). In our study, gender was identified as one of the predictive factors, with the incidence of APTB being higher in men than in women. Previous studies have shown that MCV is a marker of pulmonary inflammation (28). In patients with APTB, MCV levels are negatively correlated with pulmonary bacterial load (29), and hypoalbuminemia is positively correlated with the severity of APTB (30). Our case group had lower levels of TC, ALB, and MCV, likely due to the high replication rate and activity of tuberculosis in active pulmonary tuberculosis patients, which leads to greater nutritional consumption. APTB patients are attacked by toxins and bacteria, which can activate immune cells in the lung tissue and release inflammatory mediators (16). This, in turn, activates platelets and promotes their adhesion and aggregation, therefore, patients with APTB have a hypercoagulable state (16). In our study, FIB levels were higher in the case group, consistent with previous research findings. When an inflammatory response occurs, the counts of white blood cells and neutrophils in the blood significantly increase (31). Our control group consisted of bronchiectasis patients with other infections, primarily bacterial pneumonia, at our hospital. Therefore, compared to the case group, the control group had higher LYM levels. Controversially, few studies have demonstrated that ESR and CRP are sensitive markers for tuberculosis (32). However, hematological parameters such as hemoglobin, PCV, RBC count, blood indices, platelet count, WBC count, and ESR can still be utilized for diagnosis, prognosis, and follow-up of patients (33). Inflammatory biomarkers like MLR, CRP, and hs-CRP have been shown to be associated with TB diagnosis (18). CRP, in particular, may be a reliable indicator for diagnosing TB co-infected with other infections (34).

Nomograms are widely used for predicting tumors and various diseases due to their user-friendly digital interface, high predictive accuracy, and ease of understanding (35, 36). Compared to traditional prediction models, they facilitate more rational clinical decision-making (37, 38). The model calculates the risk of APTB, providing insights and methods for its early prevention. The higher the nomogram score, the greater the risk of developing APTB (C-index of 0.745; 95% CI: 0.715–0.775). The established nomogram model was validated using ROC and calibration curves, demonstrating that the predicted probability of APTB risk closely matches the actual occurrence (AUC = 0.744).

However, this study has some limitations: Firstly, since the participants in this study were aged over 18 years, the performance of the diagnostic model in individuals aged under 18 years remains unclear; the data were sourced from only two hospitals, which may introduce selection bias; the retrospective nature of the study may also introduce potential biases in data collection, such as missing or incomplete clinical information. Secondly, although we carefully screened the study subjects and employed multivariate logistic regression to account for confounding variables, potential confounding effects may still persist. Further studies with more homogenous control groups are needed to validate our findings. Overall, the model demonstrates strong clinical utility and can serve as an effective tool for early screening or auxiliary diagnosis, particularly in economically underdeveloped regions or primary healthcare settings.

## 5 Conclusion

This study developed a nomogram predictive model with good accuracy and clinical applicability that consists of seven indicators: age, gender, ALB, MCV, FIB, hemoptysis, and hypertension. This model could be valuable in assessing the risk of APTB in patients with bronchiectasis.

## Data Availability

The original contributions presented in the study are included in the article/supplementary material, further inquiries can be directed to the corresponding authors.
